# Perspectives and mechanisms for targeting ferroptosis in the treatment of hepatocellular carcinoma

**DOI:** 10.3389/fmolb.2022.947208

**Published:** 2022-08-16

**Authors:** Lanqing Li, Xiaoqiang Wang, Haiying Xu, Xianqiong Liu, Kang Xu

**Affiliations:** ^1^ Department of Otolaryngology-Head and Neck Surgery, The First Affiliated Hospital of Chongqing Medical University, Chongqing, China; ^2^ Hubei Engineering Technology Research Center of Chinese Materia Medica Processing, College of Pharmacy, Hubei University of Chinese Medicine, Wuhan, China

**Keywords:** ferroptosis, hepatocellular carcinoma, cell death, molecular signaling, targeted therapy

## Abstract

Ferroptosis is a novel process of regulated cell death discovered in recent years, mainly caused by intracellular lipid peroxidation. It is morphologically manifested as shrinking of mitochondria, swelling of cytoplasm and organelles, rupture of plasma membrane, and formation of double-membrane vesicles. Work done in the past 5 years indicates that induction of ferroptosis is a promising strategy in the treatment of hepatocellular carcinoma (HCC). *System xc* ^*-*^ */GSH/GPX4*, iron metabolism, p53 and lipid peroxidation pathways are the main focus areas in ferroptosis research. In this paper, we analyze the ferroptosis-inducing drugs and experimental agents that have been used in the last 5 years in the treatment of HCC. We summarize four different key molecular mechanisms that induce ferroptosis, i.e., *system xc* ^*-*^ */GSH/GPX4*, iron metabolism, p53 and lipid peroxidation. Finally, we outline the prognostic analysis associated with ferroptosis in HCC. The findings summarized suggest that ferroptosis induction can serve as a promising new therapeutic approach for HCC and can provide a basis for clinical diagnosis and prevention of this disease.

## Introduction

Cell death is important for maintaining homeostasis and cellular functions. Cell death is classified into two types: accidental cell death (ACD) and regulated cell death (RCD) (Fuchs and Steller, 2011). Of these, RCD is further divided into apoptosis, necroptosis, pyroptosis, and ferroptosis in the early stage (Grootjans et al., 2017; Tang D et al., 2019). Different types of cell death have different morphological characteristics and response mechanisms with distinct molecular mechanisms and regulatory factors. Ferroptosis as a mechanism of cell death was first proposed in 2012. It is an iron-dependent form of RCD that, unlike apoptosis, is not dependent on caspases or the BCL-2 family. It is mainly characterized by accumulation of ferrous ions and unrestricted lipid peroxidation leading to plasma membrane rupture (Dixon et al., 2012; Stockwell et al., 2017).

Liver diseases are the leading cause of death worldwide (Asrani et al., 2019; Fu et al., 2022). Liver is the metabolic center for absorption of glucose, amino acids, and other nutrients (Feng F et al., 2021; Pan et al., 2021; Li L et al., 2022; Pan et al., 2022). Therefore, dysregulation of liver function may lead to oxidative stress, potentially causing a series of liver diseases. Ferroptosis plays an important role in liver metabolic pathways such as regulation of NADPH levels, GSH levels, and fatty acid metabolism (Chen et al., 2022). In addition, iron metabolism, which induces ferroptosis, is also mainly regulated by the liver. Ferroptosis-induced cell death plays an important role in the development of liver diseases such as hemochromatosis, alcohol-associated liver disease (ALD), hepatitis C virus (HCV) infection, non-alcoholic steatohepatitis (HCV), and hepatitis B, alcoholic steatohepatitis (NASH), and hepatocellular carcinoma (HCC) (Nie et al., 2018; Shojaie et al., 2020).

The latest cancer statistics released in 2020 in the Global Cancer Statistics Report show that primary liver cancer is the sixth most common cancer and the third most deadly cancer worldwide (Sung et al., 2021). HCC ranks fifth in global incidence and places a significant economic burden on the world’s public health systems. Owing to the rapid growth and migration of HCC, it is often difficult to control its development with existing treatment modalities, ultimately leading to a lower survival rate (Chen et al., 2021b; Li L et al., 2022). Modern HCC treatment include both surgical and non-surgical treatments. The proportion of patients that can be treated surgically is less than 30% (Zhou et al., 2016). Furthermore, surgical treatment is associated with a 40% recurrence rate (Ryerson et al., 2016). Non-surgical treatments include targeted drug therapy (Liu Z et al., 2019; Song et al., 2019), chemotherapy (Goyal et al., 2019), radiotherapy (Zaheer et al., 2019), and Chinese medicine (Yang et al., 2020b). These treatment strategies are aimed at selective killing of cancer cells without affecting normal cells. Despite this, existing treatments often have the disadvantages of also causing normal cell death along with incomplete killing of cancer cells. In recent years, a major breakthrough has been made in inhibiting the growth of HCC by selective induction of cell death.

Recent studies have shown that the tumor microenvironment plays a complex and multifaceted role in the induction of cancer cell ferroptosis. For example, immune cells such as neutrophils and macrophages can be recruited into cancer tissue through chemokines released from cancer cells and cancer-associated stromal cells. These are then directed by proteins, metabolites, etc., to perform pro- or anti-tumor functions, thus affecting the regulation of iron metabolism in cancer cells (Liang and Ferrara, 2020). In addition, substantial progress has been made in the treatment of HCC *via* targeted regulation of ferroptosis. The classical method of induction of ferroptosis is by blocking intracellular glutathione peroxidase GPX4 through the inhibition of cystine/glutamate transporter (system xc^−^), thus resulting in inhibition of HCC cell proliferation (Yang et al., 2014). Modern drugs used to induce the onset of ferroptosis in liver cancer cells are erastin and sorafenib, which target the RCD process and may provide a new effective therapeutic measure to inhibit HCC.

In this paper, we present a systematic summary of the mechanisms of ferroptosis, including System xc^−^, iron metabolism, p53, and lipid peroxidation. We also enumerate the drugs and novel technologies used to target ferroptosis in recent years and discuss how ferroptosis can be used as a target in liver cancer treatment. Finally, we highlight several key questions and challenges for future research.

## Ferroptosis overview

**FIGURE 1:**
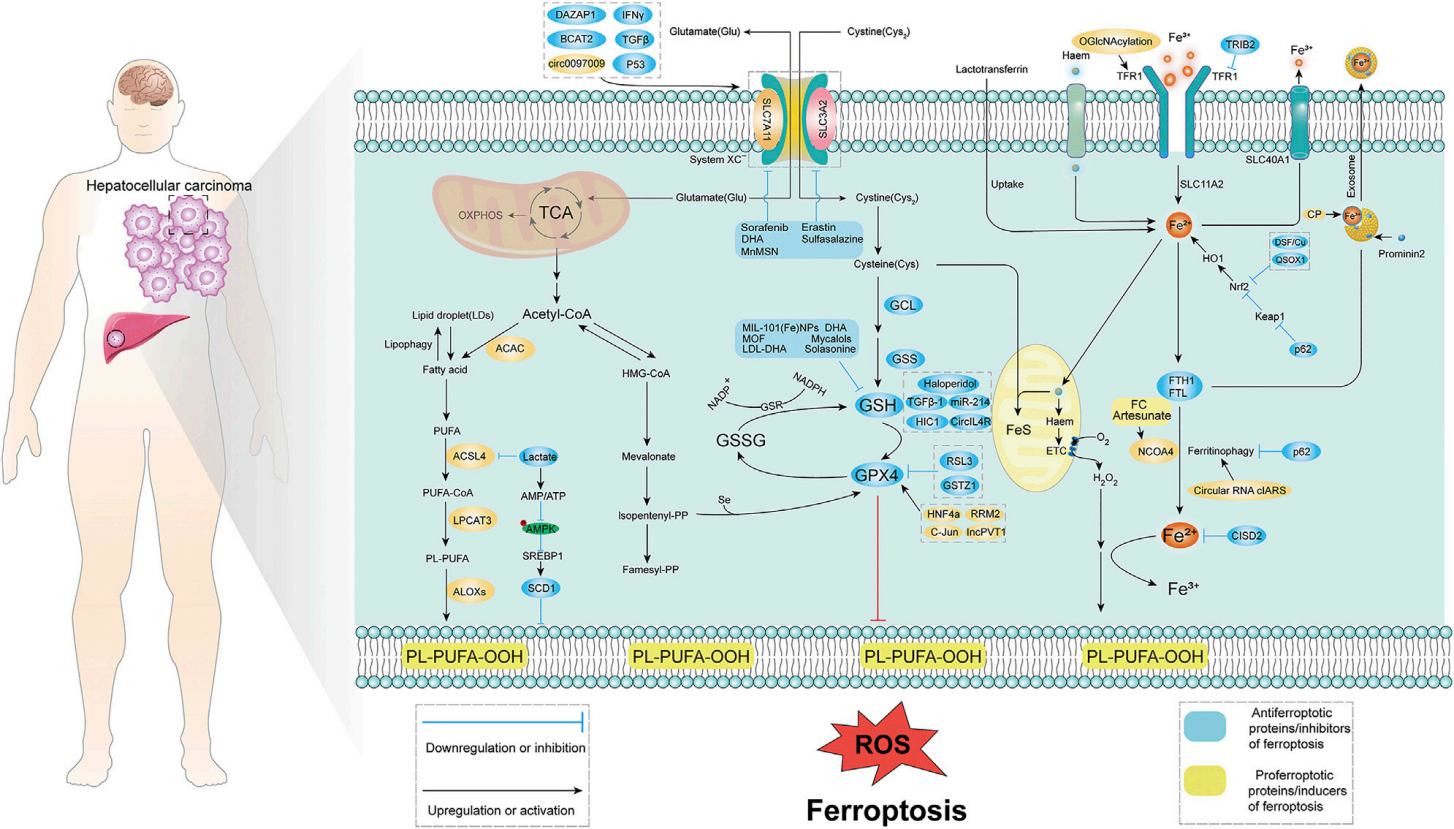
Molecular mechanism of ferroptosis in HCC. Ferroptosis is mainly caused by lipid peroxidation, and proper induction of ferroptosis may be an effective treatment for related cancers. The occurrence of ferroptosis mainly involves three aspects: System xc^−^, iron metabolism, p53, and lipid peroxidation. System xc^−^ introduces cystine into cells primarily at a 1:1 reverse amino acid transport ratio, ultimately in glutamate-cysteine ligase (GCL), glutathione synthase (GSS), glutathione Induced intracellular lipid peroxidation under the action of peptide peroxidase. Iron metabolism is mainly caused by lipid peroxidation caused by excessive release of iron ions with the participation of iron ions. Lipid peroxidation is mainly through fatty acid-induced lipid peroxidation. TCA, tricarboxylic acid cycle; ROS, reactive oxygen species; GSH, glutathione; GPX4, glutathione peroxidase; TFR1, transferrin receptor; Acetyl-CoA, acetyl-CoA; PUFA, poly unsaturated fatty acid.

Ferroptosis, a unique Fe-dependent cell death mechanism, was first proposed in 2012. Cells undergoing ferroptosis exhibit distinct morphological features such as shrunken mitochondria and reduced number of mitochondrial ridges. It is mainly characterized by an excessive accumulation of lipid peroxide leading to impaired cell membrane function (Dixon et al., 2012; Stockwell et al., 2017) (Figure 1). Ferroptosis can be induced by inhibition of cell membrane transport proteins through an exogenous pathway or by blocking the activation of intracellular antioxidant enzymes (Tang and Kroemer, 2020). Ferroptosis induces a unique form of cell death that offers a potential for developing novel drugs for cancers that are difficult to treat with conventional therapies. Induction of ferroptosis in HCC is an attractive alternative novel therapeutic approach for liver cancer. We have compiled relevant physiological studies describing ferroptosis in HCC and describe the relevant targets of action and mechanisms (Table 1).

**TABLE 1:** Pathways and targets of ferroptosis in HCC.

Target	Effector/reagent	Proposed mechanism	References
SLC7A11	Inhibition of DAZAP1	Destabilization of SLC7A11	Choudhury et al. (2014); Chen et al. (2020); Wang et al. (2021d)
	Knock down circ0097009	Downregulation of SLC7A11 expression	Lyu et al. (2021)
SLC7A11 and SLC3A2	IFNγ	IFNγ activates JAK/STAT and downregulates SLC7A11 and SLC3A2 expression	Kong et al. (2021)
System Xc^-^	Inhibition of BCAT	Reduces glutamate synthesis and affects System xc^-^	Wang et al. (2021c)
	TGFβ-1	Inhibition of System xc^−^, reduction of GSH and GPX4 by Smad3	Dituri et al. (2019); Kim et al. (2020)
GSH	Inhibition of C-Jun	Inhibition of GSH expression level	Vogt (2002); Chen Y et al. (2019)
	Inhibition of RRM2		Duxbury et al. (2004); Duxbury and Whang (2007)
	Inhibition of PSAT1		Tang H et al. (2019)
GPX4	Knockdown CircIL4R	Inhibition of GPX4 expression levels	Yao et al. (2019); Xu et al. (2020)
ferroptosis	Upward HBA1	Direct induction of ferroptosis	Tang H et al. (2019)
	HNF4a	HNF5a upregulates STMN1 to directly inhibit ferroptosis	
TFRC	OGlcNAcylation	Enhancement of YAP transcriptional activity and upregulation of TFRC	Zhu et al. (2021)
	TRIB2	Inhibition of TRIB2 and upregulation of TFRC	Guo et al. (2021)
LIP	RSL3	Upregulation of LIP	Asperti et al. (2021)
Iron ions	CP	CP-FPN system causes iron ion efflux and inhibits ferroptosis	Shang et al. (2020)
Ferritin autophagy	Circular RNA cIARS	Negative regulation of ALKBH5	Liu et al. (2020)
SREBP1 and SCD1	Lactate	Inhibition of HCAR1/MCT1 was able to block ATP production, which initiated AMPK phosphorylation, inhibited the expression of downstream SREBP1 and SCD1, and suppressed ferroptosis	Zhao et al. (2020)
ACSL4	Lactate	Inhibition of ACSL4, inhibition of ferroptosis	Zhao et al. (2020)
POR	G6PD	Positive regulation	Yang et al. (2019); Koppula et al. (2021)

## Hepatocellular carcinoma- related pathways in ferroptosis

### System xc^−^/GSH/GPX4

The System xc^−^/GSH/GPX4 axis plays a crucial role in promoting lipid peroxidation in the induction of ferroptosis (Galadari et al., 2017). System xc^−^ acts as a cystine and glutamate transporter in the cell and promotes intracellular GSH synthesis by simultaneously transporting cystine into and glutamate outside the cell (Capelletti et al., 2020). GSH is mainly composed of glutamate, cysteine, and glycine. These amino acids contain sulfhydryl structures that can be oxidized, allowing GSH to protect cells from oxidative stress damage. As GSH serves as a cofactor for the selenoenzyme GPX4, inhibiting the *de novo* GSH synthesis induces ferroptosis by inactivating GPX4 (Ingold et al., 2018).

System xc^−^ is a reverse transporter protein located on the plasma membrane and consists of a light chain subunit SLC7A11 (x CT) and a heavy chain subunit SLC3A2 (CD98hc or 4F2hc) linked by a covalent disulfide bond. Of these, SLC7A11 is highly specific for cystine and glutamate. SLC3A2 is a chaperone protein that helps to enhance the stability of SLC7A11 and participates in regulating the transport of SLC7A11 to the plasma membrane (Koppula et al., 2018; Lin W et al., 2020). Inhibition of System xc^−^ can effectively downregulate GSH/GPX4 expression, thereby inducing cancer cell ferroptosis. DAZAP1 is an RBP that was initially found to be abundantly expressed in the liver, heart and brain (Dai et al., 2001). Recent studies have shown that inhibition of DAZAP1 expression can significantly destabilize SLC7A11 (Choudhury et al., 2014; Chen et al., 2020; Wang et al., 2021d). Another recent study showed that downregulation of SLC7A11 expression both at mRNA and protein levels was effective in promoting ferroptosis. IFNγ is a glycosylated protein that can induce apoptosis or autophagy in tumor cells *via* immune cells along with other molecules (Castro et al., 2018; Alspach et al., 2019). IFNγ was able to sensitize HCC cells to ferroptosis by activating the JAK/STAT pathway in HCC, downregulating the mRNA and protein levels of SLC7A11 and SLC3A2, eventually inhibiting System xc^−^ activity (Kong et al., 2021). BCAT2 is a transaminase that mediates sulfur amino acid metabolism whose inhibition can reduce glutamate *de novo* synthesis, affect the conversion of System xc^−^ cystine to glutamate, and inhibit cystine uptake, thus inducing ferroptosis (Wang et al., 2021c). Transforming growth factor beta (TGFβ-1) can regulate cell growth and differentiation, and has inhibitory effects on cancer cells. TGFβ-1 can inhibit xCT through Smad3 and reduce the expression of GSH and GPX4 (Dituri et al., 2019; Kim et al., 2020). Finally, Circular RNAs (circRNAs) are a class of non-coding RNAs that can inhibit the growth, migration, and invasion of HCC (Yao et al., 2017). Indeed, knockdown of circ0097009 was found to enhance the sensitivity of HCC cells to ferroptosis through miR-1261 downregulation of SLC7A11 expression (Lyu et al., 2021).

GSH is a scavenger of free radicals and is the main cofactor involved in lipid peroxide reduction by GPX4. It has an important role in cellular defense against oxidative stress. Several studies have shown that inhibition of GSH/GPX4 can increase ferroptosis. Inhibition of O-GlcNAcylated c-Jun, the first oncogenic factor identified, can inhibit GSH synthesis by suppressing PSAT1 and CBS transcription (Vogt, 2002: Chen Y et al., 2019). Ribonucleotide reductase (RR) is essential during DNA replication and repair, and consists of two subunits (RRM1 and RRM2). RRM2 plays an important role in tumor development (Duxbury et al., 2004; Duxbury and Whang, 2007). RRM2 expression was significantly elevated in HCC, and its inhibition induced ferroptosis through GSS inhibition of GSH synthesis (Yang et al., 2020a). Ferroptosis upregulation factor (FUF) and ferroptosis downregulation factor (FDF) are regulated by transcription factors HIC1 and HNF4a respectively. HIC1 induces ferroptosis directly through upregulation of HBA1 or by suppressing the expression of PSAT1 leading to downregulation of GSH. HNF4a on the other hand inhibits ferroptosis through upregulation of STMN1 or by upregulation of PSAT1 leading to upregulation of GSH. By controlling HIC1 and HNF4a expression, it is possible to selectively inhibit GSH expression (Tang H et al., 2019). Non-coding RNAs have also been reported to be involved in tumor suppression (Liu et al., 2016). CircIL4R was shown to be significantly upregulated in HCC tissues and circIL4R knockdown was able to inhibit GPX4 activity *via* mir-541-3p (Yao et al., 2019; Xu et al., 2020). Taken together, these studies suggest that HCC can be effectively inhibited by inhibiting the System xc^−^/GSH/GPX4 axis. However, some cancer cells remain resistant to ferroptosis even after GPX4 inhibition, indicating the existence of additional ferroptosis defense mechanisms that deserve further investigation.

### TP53

As one of the “star molecules” in antitumor research, p53 has attracted the attention of researchers around the world. Its functions and post-translational modifications have highlighted the diversity and complexity of this protein (Liu Y et al., 2019). Studies have shown that deletion or mutation of the p53 gene leads to loss of wild-type p53 activity and malignant transformation of tumors (Bykov et al., 2018; Levine, 2020). Approximately 50% of patients with HCC have p53 gene deletion in their tumor cells (Aning and Cheok, 2019). Traditionally, it was thought that p53 mainly induces cell cycle arrest, senescence or apoptosis (Brady et al., 2011; Li et al., 2012; Liu and Gu, 2022). However, in recent years, it has been found that p53 can have oncogenic functions even with loss of function mutations (Liu and Gu, 2021). Several studies have shown that p53 plays a crucial role in regulating tumor metabolic activities (including glucose metabolism, oxidative phosphorylation, and lipid metabolism) (Schwartzenberg-Bar-Yoseph et al., 2004; Contractor and Harris, 2012; Liu and Gu, 2021). TP53 is also involved in regulating ferroptosis. On the one hand, p53 is able to inhibit SLC7A11 expression at the transcriptional level, increasing the likelihood of ferroptosis through GSH-dependent versus non-dependent (P53/SLC7A11/ALOX12) forms (Jiang et al., 2015; Ou et al., 2016; Venkatesh et al., 2020). On the other hand, p53 is also able to inhibit ferroptosis. For example, p53 inhibits ferroptosis by activating iPLA2β but promotes ferroptosis when external stimuli exceed a certain threshold (Chen et al., 2021a). Notably, although p53 has also been widely explored in HCC, there are very few reports highlighting its role in inducing ferroptosis in HCC. Therefore, how p53 induces or inhibits ferroptosis in HCC is a topic that needs further investigation.

### Iron metabolism

Iron is indispensable for maintaining normal life activities of organisms. Iron metabolism plays an important role in the process of ferroptosis, in which extracellular Fe^3+^ is transported to the cell and reduced to Fe^2+^ through transferrin on the cell membrane. Fe^2+^ accumulates with excess intracellular H_2_O_2_ through the Fenton reaction leading to ROS, which promotes intracellular lipid peroxide (LPO) production and triggers ferroptosis (Stockwell et al., 2017).

Ferritin is an important site for intracellular Fe^2+^ storage. Release of sufficient Fe^2+^ through autophagy induces ferroptosis. Transferrin receptor (TFRC) is a protein located on the membrane whose expression correlates with tumor stage or cancer progression. Early targeted regulation of TFRC expression has been reported as an effective strategy for the treatment of various cancers (Horonchik and Wessling-Resnick, 2008; Daniels et al., 2012). For example, OGlcNAcylation, a reversible post-translational modification catalyzed by O-GlcNAc transferase (OGT), significantly enhances YAP transcriptional activity, which leads to increased TFRC expression. TFRC overexpression enhances cellular iron uptake and enhances ferroptosis (Zhu et al., 2021). Another study indicated that Tribbles homolog 2 (TRIB2), which is highly expressed in HCC, suppresses TFRC expression, thereby inhibiting Fe^3+^ uptake and reducing ferroptosis (Guo et al., 2021). Iron ions exist in the form of divalent ions in the cytoplasm, and formation of the cytoplasmic unstable iron pool (LIP) is key to ferroptosis induction. In well-differentiated HepG2 cells, GSH peroxidase 4 inhibitor (RSL3, (1S,3R)-RSL3) treatment can upregulate LIP levels, while the levels of transferrin receptor 1 (TFR1), membrane iron transport protein 1 (FPN1), and ferritin, related proteins involved in ferroptosis, were reduced (Asperti et al., 2021). Copper cyanidin (CP) is a copper-containing glycoprotein, mainly synthesized by the liver and present in large amounts in human plasma, which can assist FPN to export iron ions and regulate iron metabolism. The CP-FPN system causes iron ion efflux and inhibits ferroptosis. Thus, downregulation of CP can induce ferroptosis (Shang et al., 2020). Circular RNAs also play a key role in iron metabolism. It was reported that the circular RNA cIARS can negatively regulate ALKBH5 (demethylase, an autophagy inhibitor) to induce ferritin autophagy and release ferric ions thereby enhancing the effect of SOR treatment-induced ferroptosis (Liu et al., 2020). Thus, iron metabolism can increase ferroptosis sensitivity by multiple mechanisms.

### Lipid peroxidation

Fatty acid components of the mevalonate pathway and membrane phospholipids are involved in ferroptosis. The mevalonate pathway is mainly dominated by Acetyl Coenzyme A (CoA), while the fatty acids of membrane phospholipids are mainly involved in polyunsaturated fatty acids (PUFA). Polyunsaturated fatty acids (PUFAs) are susceptible to oxidation in ferroptosis, leading to disruption of the lipid bilayer and affecting cell membrane function. The biosynthesis and maintenance of normal physiological functions of polyunsaturated fatty acids in cell membranes requires a series of enzymes, such as ACSL4 and LPCAT3, to ensure that the cell membrane is not disrupted.

In cancerous cells, sugar metabolism is at the core of energy generation, and its metabolic characteristics are distinct from normal cells. Tumor cells mainly use glycolysis as the mode of energy production, i.e., Warburg effect (Hanahan and Weinberg, 2011). Lactate is a more involved product in Glucose metabolism, and studies have shown that lactate uptake by cancer cells is mainly achieved through the transporter protein MCT1, whose expression level is regulated by HCAR1 (Roland et al., 2014; Khan et al., 2020; Tasdogan et al., 2020). Excess lactate is able to support ATP production through the tricarboxylic acid cycle (TCA), and by inhibiting HCAR1/MCT1 is able to hinder ATP production. This initiates AMPK phosphorylation, inhibits downstream SREBP1 and SCD1 expression, and induces ferroptosis. In addition, lactate inhibits the expression of ACSL4 and thus protects HCC cells from ferroptosis. Therefore, blocking lactate uptake may also induce ferroptosis and inhibit HCC (Zhao et al., 2020).

Glucose-6-phosphate dehydrogenase (G6PD) was shown to be a key enzyme in the pentose phosphate pathway (PPP) and plays a critical role in the production of NADPH. G6PD positively regulates ferroptosis by regulating POR (Cao et al., 2021). Cytochrome P450 oxidoreductase (POR) increases ferroptosis by upregulating peroxidation of membrane polyunsaturated phospholipids (Yang et al., 2019; Koppula et al., 2021), In conclusion, metabolic pathways are able to participate in the induction of ferroptosis in cancer cells to varying degrees. However, HCC is not well studied in the context of lipid peroxidation-induced ferroptosis, a potential pathway to induce ferroptosis in HCC.

## Current stage of pharmacological study of ferroptosis in hepatocellular carcinoma

**FIGURE 2:**
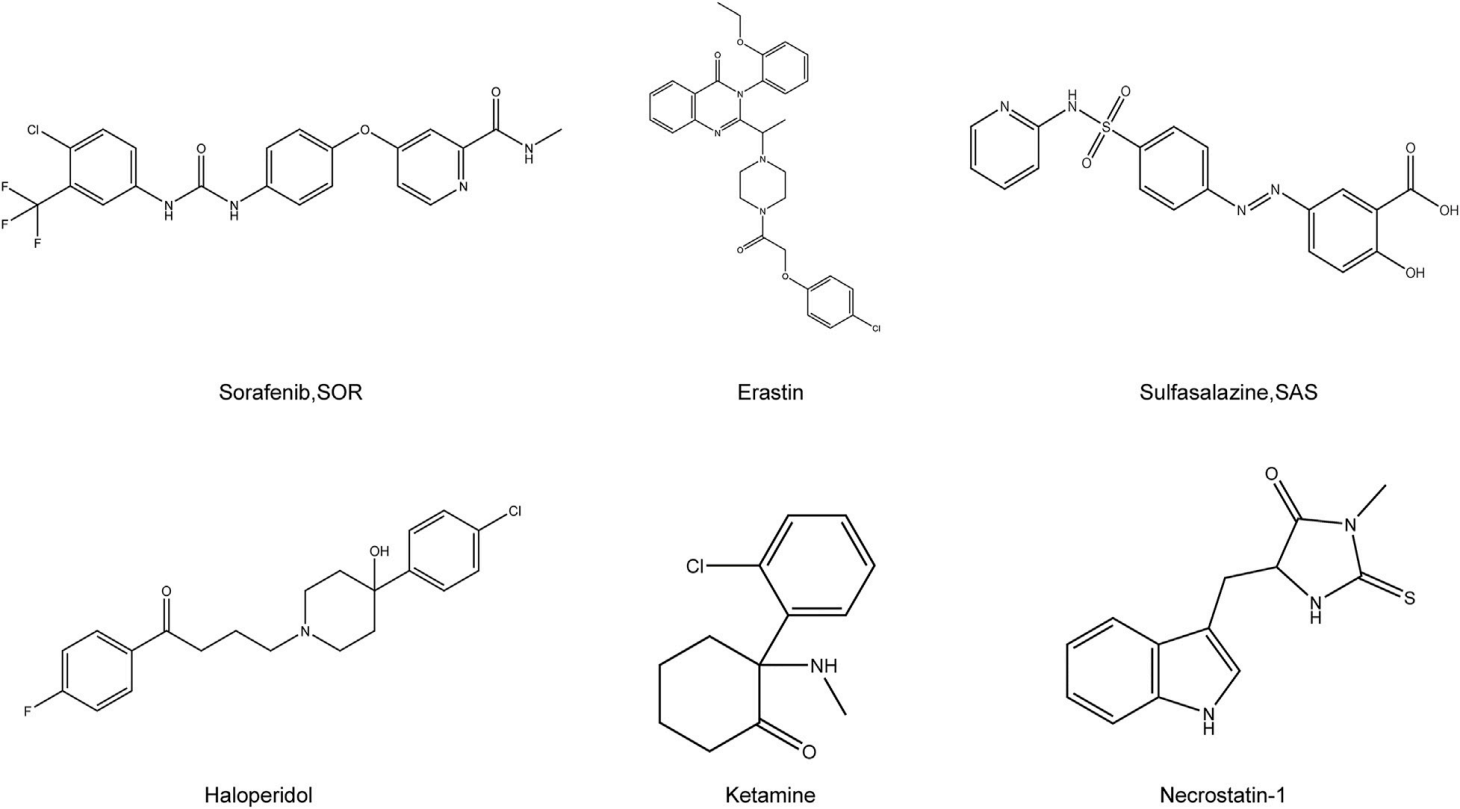
Chemical structure of drugs related to the treatment of HCC by ferroptosis. Structural formula of some compounds that can target ferroptosis to inhibit HCC. Sorafenib, SOR; Sulfasalazine, SAS.

The recent discovery of ferroptosis has led to its application in the inhibition of liver tumors. Use of ferroptosis inducers and emerging technologies offers new possibilities for treating patients with HCC with potentially reduced side effects (Figure 2). In the next few sections, we describe the signals associated with ferroptosis in HCC and highlight the potential therapeutic agents for clinical translation (Table 2).

**TABLE 2:** Drugs and pathways of action related to ferroptosis in HCC.

Target	Remarks	Action mechanism	References
SOR		Disruption of mitochondrial morphology and reduction of ATP synthesis	Li Y et al. (2021)
		GSH consumption	
	Knockdown MT1G	Increase GSH consumption	Houessinon et al. (2016); Sun et al. (2016a)
	SPARC overexpression	Promotes LDH release andenhances the toxic effects of SOR	Hua et al. (2021)
	Joint use of RSL3	Inhibition of GSTZ1 increases the effect of SOR on the induction of ferroptosis	Wang K et al. (2021)
	Downward adjustment of Rb	Increased SOR-induced mortality in HCC cells	Louandre et al. (2015)
	Knock down CISD2	Increase ROS,MDA levels and promote SOR-induced ferroptosis	Li B et al. (2021)
	Reduce P62, NRF2	Promote SOR-induced ferroptosis	Ichimura and Komatsu (2018)
	DSF/Cu,NRF2	Promote SOR-induced ferroptosis	Ren et al. (2021)
	QSOX1,NRF2	Promote SOR-induced ferroptosis	Sun et al. (2021)
Natural Products	Formosanin C	Downregulation of ferritin heavy chain polypeptide 1 (FTH1) and upregulation of NCOA4	Lin P. L et al. (2020)
	Heteronemin	Induction of ROS formation	Chang et al. (2021)
	Mycalols	Decrease the expression of GPX4 and increase the expression of NCOA4	Riccio et al. (2021)
	Solasonine	Inhibition of Gpx4 and GSS	Jin et al. (2020)
	Artesunate	Promotes lysosomal histone B/L activation, ferritin autophagy, and lipid peroxidation	Li Z. J et al. (2021)
	DHA	Increased expression of ROS, MDA and decreased activity of glutathione (GSH), GPX4, solute carrier family (SLC) SLC7A11, SLC3A2	Wang Z et al. (2021)
	*S. barbata*	Decreased GPX4 and SLC7A11, increased IREB2, ACSL4 expression	Li Y et al. (2022)
	Atractylodin	Decrease GPX4,FTL levels and increase ACSL4,TFR1 levels	He et al. (2021)
Novel Drug Technologies	MnMSN(FaPEG-MnMSN@SFB)	Depletion of HSG, suppression of System xc^-^	Tang H et al. (2019); Tang H et al. (2020)
	HKUST-1	Integrates cyclooxygenase 2 (COX-2), which depletes GSH and inhibits GPX4 activity; induces PINK1/Parkin-mediated mitochondrial autophagy	Gu et al. (2011); Tian et al. (2022)
	MIL-101(Fe)@sor NPs	Increased lipid peroxidation and MDA levels, decreased GSH and GPX4	Liu et al. (2021)
	Cas13a or microRNA combined with iron nanoparticles	Induce ferroptosis	Sun et al. (2021)
	LDL-DHA	GSH depletion and inhibition of GPX4	Ou et al. (2017)
	Exosomes (ExosCD47)	Bypassing the phagocytic effects of MPS, chemophotodynamic therapy targets the induction of ferroptosis	Chen R et al. (2019); Du et al. (2021)
Others	MicroRNA-214-3p (miR-214) combined with Erastin	Increased MDA, ROS expression levels, upregulated iron ion concentration, and decreased GSH levels	Bai et al. (2020)
	Ketamine	Inhibits GPX4 expression	Zhu et al. (2021)
	Haloperidol	Downregulation of S1R, downregulation of GSH levels and upregulation of lipid peroxidation in liver cancer cells	Wang et al. (2015); Bai et al. (2019)
	Nucleoprotein 1 (NUPR1) inhibitor ZZW-115	Disruption of mitochondrial morphology and metabolic function	Huang et al. (2021)
	YAP/TAZ	Induces the expression of SLC7A11 and inhibits ferroptosis	Gao et al. (2021)
	Necrostatin-1	Induces the expression of SLC7A11 and inhibits ferroptosis	Yuk et al. (2021)

### Sorafenib

Sorafenib (SOR) is the first multi-tyrosine kinase inhibitor approved for the treatment of patients with unresectable HCC, advanced kidney cancer, and differentiated thyroid cancer (Kim and Park, 2011; Zhu et al., 2017). The main feature of this compound is its ability to not only directly inhibit tumor cell proliferation but also indirectly inhibit tumor angiogenesis. Although several cancer-associated protein kinase targets have been identified for SOR in HCC, the underlying mechanism of its action remains unclear. Despite its potential, clinical SOR use is associated with side effects. These include worsening liver dysfunction and reduced survival benefit in SOR-treated patients with advanced cirrhosis of Child-Pugh class B or C (Pinter et al., 2009; Wörns et al., 2009). Although SOR has been consistently reported to cause ferroptosis *via* system xc^−^, its mechanism was unlike the homologous system xc^−^ inhibitors sulphasalazine and elastin. Therefore, SOR is not exclusively responsible for HCC cell death *via* system xc^−^ induced ferroptosis in different HCC cell lines (Zheng et al., 2021).

It was shown that the anticancer activity of SOR *via* ferroptosis induction relies mainly on the inhibition of System xc^−^ (Dixon et al., 2014). Therefore, we will discuss the specific mechanism of SOR-mediated induction of ferroptosis in HCC. SOR-mediated inhibition of liver cancer cell growth is not *via* a single apoptotic process. Studies have revealed that blocking apoptosis does not prevent the iron-dependent cytotoxicity of SOR. In contrast, iron chelation did not prevent the toxic effects of SOR on HCC cells under pro-apoptotic conditions. These results suggest that SOR may be a better inducer of ferroptosis in cultured liver cancer cells (Louandre et al., 2013). Treatment of HCC cells with SOR for different times produced different levels of phosphorylation in HCC cells. E3 ubiquitin protein ligase MDM2 (Q00987) is involved in p53 regulation. The phosphosite pSer166 (FC = 0.16, *p* = 0.022) on MDM2 is a key residue in this regulation. Sorafenib treatment resulted in sixfold reduction of pSer166 levels, while pSer315 (FC = 0.25, *p* = 0.019) and pSer392 (FC = 0.02, *p* = 0.027) sites on p53 decreased significantly by 4-fold and 50-fold, respectively. By 60 min, significant changes were observed in p53 (P04637), CAD protein (P27708), and iron homeostasis important proteins such as heavy chain ferritin FTH1, heme oxygenase 1 (HMOX1; P09601), and PCBP1 (Q15365). These key targets are largely correlated with ferroptosis, suggesting a possible involvement of phosphorus-regulated signaling during SOR-induced ferroptosis (Werth et al., 2020). In addition, ferroptosis manifests morphologically as disrupted mitochondrial morphology. SOR is thus able to disrupt the mitochondrial morphology of HCC cells accompanied with decreased oxidative phosphorylation activity, mitochondrial membrane potential and ATP synthesis, and subsequent cell death *via* ferroptosis. In addition, depletion of glutathione through cysteine deprivation or cysteinase inhibition exacerbates SOR-induced ferroptosis and lipid peroxide production, enhances oxidative stress and mitochondrial ROS accumulation, and induces ferroptosis (Li Y et al., 2021). However, SOR resistance is a potential problem in the treatment of patients with HCC. New findings suggest that NRF2 activation upregulates the expression of MT1G mRNA of the metallothionein-1 (MT1) family during SOR treatment. Knockdown of MT1G increases glutathione (GSH) depletion and lipid peroxidation, thus inducing ferroptosis. Therefore, MT1G may be a key regulator to target in tackling drug resistance during SOR chemotherapy (Sun et al., 2016a; Houessinon et al., 2016).

In HCC, there are some regulators can synergize or antagonize SOR action. For instance, overexpression of cysteine-rich secretory acidic protein (SPARC) induces oxidative stress, which induces ferroptosis. This promotes the release of lactate dehydrogenase (LDH), disrupts the expression of proteins associated with ferroptosis, and enhances the toxic effects of SOR in Hep3B and HepG2 cells (Hua et al., 2021). Glutathione s-transferase (GSTZ1), an enzyme involved in phenylalanine metabolism, is significantly downregulated in SOR-resistant HCC cells. Downregulation of GSTZ1 leads to activation of the NRF2 pathway, which upregulates glutathione peroxidase (GPX4) and inhibits ferroptosis. The GPX4 inhibitor RSL3 significantly inhibits GSTZ1 and promotes ferroptosis. Thus, the use of RSL3 may provide a new therapeutic strategy for HCC (Wang K et al., 2021). Loss of function of the Retinoblastoma (Rb) protein has an important effect on hepatocarcinogenesis. Thus, by downregulating Rb levels, the mortality rate in SOR-exposed HCC cells is two to three times higher than with SOR alone (Louandre et al., 2015). The mitochondrial outer membrane protein CDGSH iron-sulfur cluster structural domain 2 (CISD2) is highly expressed in HCC cells. Knocking down CISD2 expression can increase ROS, MDA, and iron ion levels, which can promote SOR-induced ferroptosis in HCC resistant cells. Thus, SOR combined with CISD2 inhibition has therapeutic potential in HCC (Li B et al., 2021). Several other studies have shown that NRF2 plays a key role in enhancing SOR-induced ferroptosis in Iron metabolism. The autophagy receptor protein p62 was found to initiate autophagy *via* the Keap1-Nrf2 signaling pathway (Ichimura and Komatsu, 2018). p62 competes with Nrf2 to bind Keap1, leading to dissociation of Nrf2 from Keap1. Thus, when p62 expression increases, Keap1 can no longer bind to Nrf2, leading to increased Nrf2 signaling and inhibition of ferritin autophagy. The expression of FTH1, HO-1, etc., is upregulated and ROS production is inhibited, thus protecting cells from ferroptosis. Therefore, inhibition of NRF2 expression or activity increases the anticancer activity of erastin and SOR *in vitro* and *in vivo* (Sun et al., 2016b). Disulfiram (DSF) is a divalent metal ion chelator that binds metal ions *in vivo* and inhibits acetaldehyde dehydrogenase activity (ALDH). Recent studies have shown DSF to possess antitumor activity, which can be enhanced in combination with Cu plasma (Skrott et al., 2017). DSF/Cu can inhibit nuclear translocation of Nrf2 and enhance SOR-induced ferroptosis to inhibit HCC cell proliferation (Ren et al., 2021). Resting sulfhydryl oxidase-1 (QSOX1) promotes the formation of disulfide bonds in peptides and proteins and also the oxidation of reduced molecules to generate hydrogen peroxide. QSOX1 is highly expressed in a variety of cancer tissues (Lake and Faigel, 2014) and studies have pointed to QSOX1 as a potential oncogene in HCC (Zhang et al., 2019). However, its expression varies in different tumor environments. QSOX1 inhibits EGF-induced EGFR activation by promoting ubiquitination-mediated EGFR degradation and accelerating its intracellular endosomal transport, resulting in reduced NRF2 activity. In addition, QSOX1 enhances sorafenib-induced ferroptosis by inhibiting NRF2 *in vitro* and *in vivo* (Sun et al., 2021). In summary, SOR plays a role in inducing ferroptosis in HCC, either alone or in combination, and to some extent can also induce ferroptosis and cause death of HCC. However, its use in the treatment of HCC is still a great challenge due to the associated side effects.

### Natural products

**FIGURE 3:**
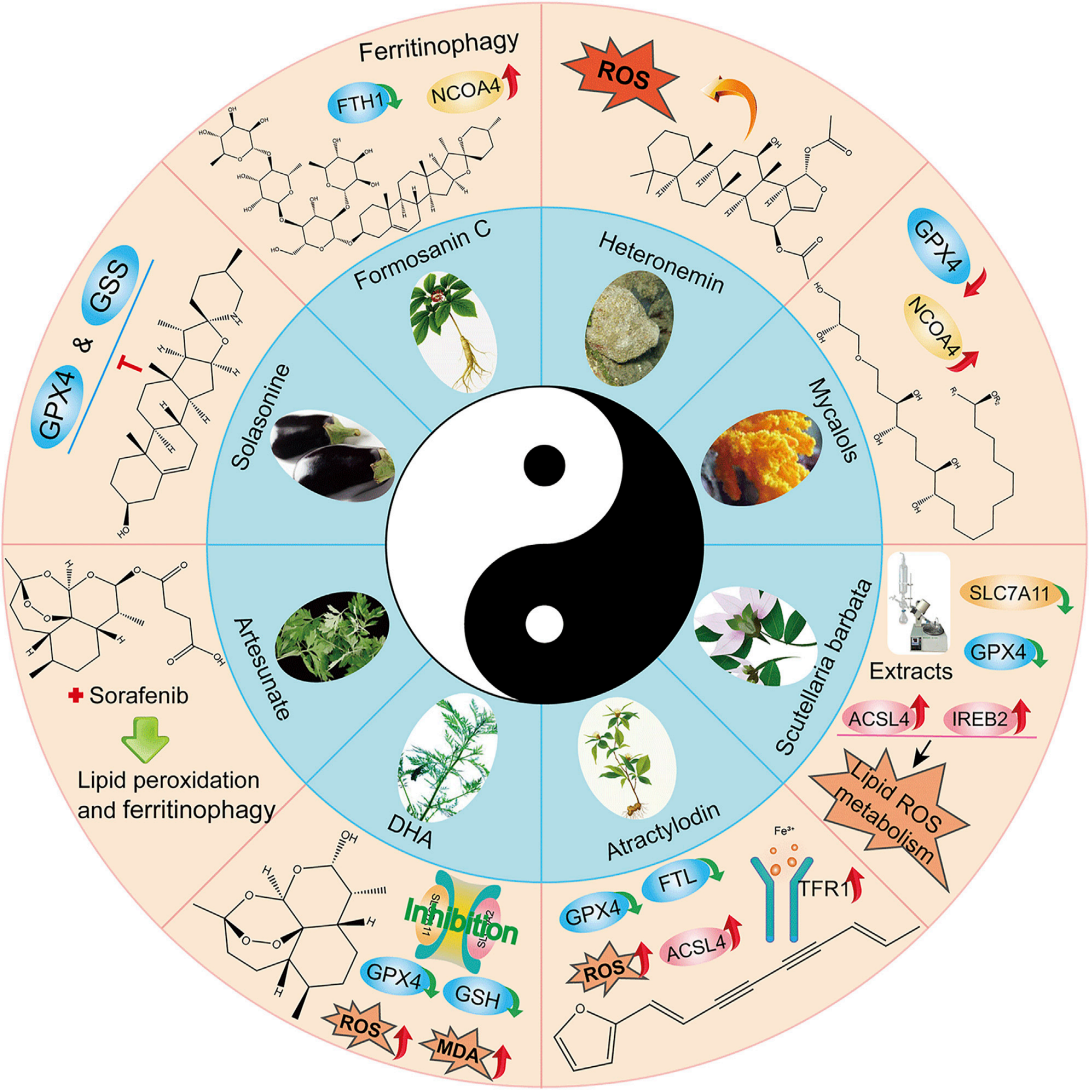
Application of Chinese medicine in the induction of HCC ferroptosis. Traditional Chinese medicine: Formosanin C(FC), Heteronemin, Mycalols, Solasonine, *S. barbata*, Atractylodin, Artesunate, Dihydroartemisinin (DHA). Of these, seven derivatives of Mycalols have been identified.

Natural plant (Fueki et al., 2022; Huang et al., 2022; Otsuki et al., 2022) extracts and plant monomers occupy a significant proportion of the research on anti-HCC compounds. Of these, herbal medicine is a focus of research (Wang et al., 2020; Wang et al., 2021a; Wang et al., 2021b) involving extracts and compounds implicated in the induction of ferroptosis to inhibit HCC (Figure 3). Several studies have shown that plant extracts and compounds are able to induce intracellular ROS production and increase susceptibility of cells to ferroptosis. For example, Formosanin C (FC), a natural compound that induces autophagic flux, inhibits HCC growth by downregulating ferritin heavy chain polypeptide 1 (FTH1), and upregulating NCOA4 expression. This causes increased ferritin autophagy, leading to increased intracellular ferric ion levels, thus increasing reactive oxygen species (ROS) levels and inducing ferroptosis (Lin P. L. et al., 2020). Heteronemin is a marine natural product isolated from the sponge *Hyrtios* sp. Heteronemin induces ROS formation and leads to p38/jnk activation and caspase-related apoptosis and ferroptosis, thereby inducing death in liver cancer cells (Chang et al., 2021). Mycalols, polyoxyglycerol alkyl ethers are mainly isolated from the Antarctic sponge *M. (Oxymycale) acerata*. Mycalol can reduce the expression level of GPX4 and increase the expression of NCOA4 (Table 3). Enhanced NCOA4 expression induces ferroptosis, which may be directly related to the inhibition of liver cancer cell growth by mycalol (Riccio et al., 2021). Solasonine, a compound isolated from Solanum melongena, has anti-infective and neurogenesis-promoting effects. Metabolomics analysis showed that Solasonine increases lipid ROS levels in HepG2 cells by inhibiting Gpx4 and GSS. Solasonine also promotes ferroptosis in HCC cells through Gpx4-induced disruption of the glutathione redox system (Jin et al., 2020). In addition, numerous Chinese herbs also possess anti-hepatocellular carcinogenic ability. For example, Semen (*Scutellaria barbata*) can increase the expression of iron peroxidation-related genes IREB2 and ACSL4 by significantly reducing the expression levels of GPX4 and SLC7A11 in nude mice. It also promotes iron peroxidation and lipid ROS metabolism to induce ferroptosis in HCC cells (Li Y et al., 2022). Atractylodin decreases GPX4 and FTL protein expression, upregulates ACSL4 and TFR1 protein expression, and increases ROS levels in liver cancer cells (He et al., 2021). In recent years, several studies have reported that active components of Artemisia annua can induce ferroptosis and inhibit HCC. Of these, artesunate, a semisynthetic derivative of artemisinin, has earlier been reported to have anticancer activity (Efferth et al., 2001), Newer studies have shown that artesunate-induced lysosomal activation synergizes with sorafenib-mediated pro-oxidation by promoting lysosomal histone protease B/L activation, ferritin autophagy, lipid peroxidation, and subsequent ferroptosis. A series of responses were significantly exacerbated by combining Artesunate and sorafenib treatments in the inhibition of HCC (Li Z. J et al., 2021). Another study showed that the artemisinin derivative dihydroartemisinin (DHA) can increase the expression of ROS, MDA, decrease the activity of glutathione (GSH), GPX4, solute carrier family (SLC) SLC7A11, SLC3A2, reduce their expression and induce ferroptosis. In addition, this study also found that DHA was able to enhance the activity of GSH degrading cation transporter-like protein 1 (CHAC1) promoter. This enhanced activity was found to influence theunfolded protein response (UPR) resulting in reduced GSH activity and inducing ferroptosis (Wang Z et al., 2021).

**TABLE 3:** Seven derivatives of Mycalols.

Mycalol-550	R_1_ = C_5_H_11_	R_2_ = Ac
Mycalol-522	R_1_ = C_3_H_7_	R_2_ = Ac
Mycalol-578	R_1_ = C_7_H_15_	R_2_ = Ac
Mycalol-594	R_1_ = C_5_H_11_	R_2_ = (3S)-3HB
Mycalol-622	R_1_ = C_7_H_15_	R_2_ = (3S)-3HB
Mycalol-636	R_1_ = C_8_H_17_	R_2_ = (3S)-3HB
Mycalol-650	R_1_ = C_9_H_19_	R_2_ = (3S)-3HB

### Novel drug technologies

**FIGURE 4:**
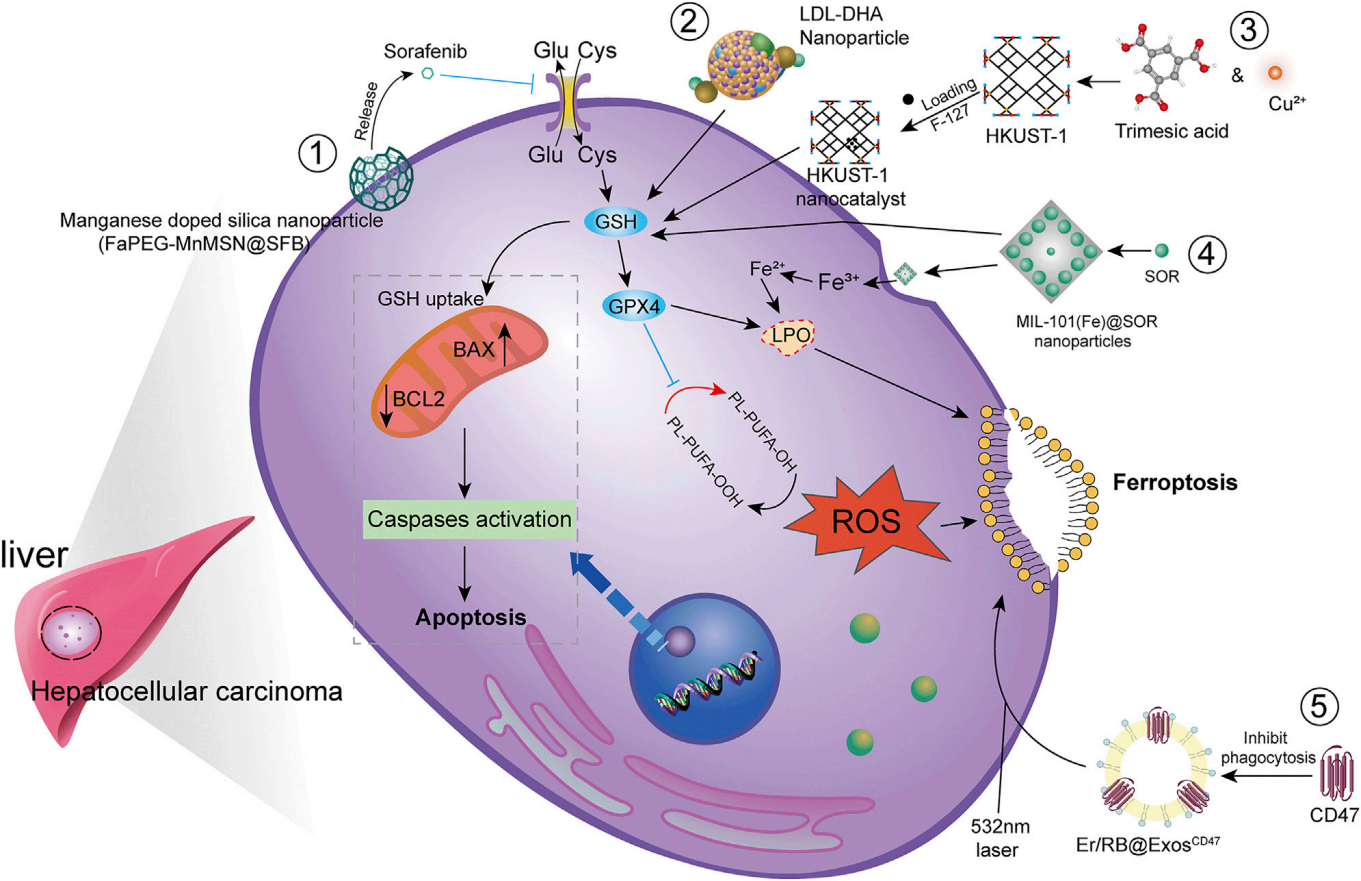
Modern technology for the treatment of HCC by ferroptosis. To achieve higher bioavailability by designing nano-encapsulated materials to prevent drug degradation, including 1) silica nanoparticles; 2) low-density lipoprotein nanoparticles; 3) metal-organic framework compound materials MOF + nanocatalyst HKUST-1; 4) iron-containing Ionic MIL-101(Fe)NPs. In addition, chemophotodynamic therapy 5) combined with exosome use induces ferroptosis.

HCC is difficult to detect in the early stages and is often transformed into advanced HCC when diagnosed clinically. Since advanced HCC often has strong resistance to chemotherapy, the efficacy of common chemotherapeutic drugs is not satisfactory. Additionally, clinical application is somewhat limited due to poor solubility, low bioavailability, and rapid metabolism of many drugs. Therefore, there is an urgent need to develop new technologies to improve bioavailability of drugs and to achieve better therapeutic outcomes (Figure 4).

Studies have shown that new technologies such as designed nanoparticles and chemical photodynamic therapy can induce ferroptosis to inhibit HCC. Biodegradable silica nanoparticles are inorganic nanoparticles that can be renally cleared and have features such as reduced toxic accumulation *in vivo* along with the advantage of facilitated drug delivery and controlled release. It was found that GSH can be depleted by developing manganese doped silica nanoparticles (MnMSN). Targeted SFB loaded MnMSN (FaPEG-MnMSN@SFB) can deplete GSH on one hand and inhibit System xc^−^ on the other hand to achieve dual inhibition of GSH and thus induce ferroptosis (Tang H et al., 2019; Tang H et al., 2020). Metal-organic framework compound material (MOF) is a highly crystalline inorganic-organic hybrid porous material. HKUST-1 nanocatalyst is a type of MOF with large surface area, high porosity, and uniform pore size (Gu et al., 2011). By integrating cyclooxygenase 2 (COX-2), inhibitor was able to deplete GSH, inhibit GPX4 activity, and trigger chemodynamic therapy (CDT) mediated ROS accumulation lipid peroxides (LPO) induced ferroptosis. In addition, COX-2 downregulation can also induce PINK1/Parkin mediated mitochondrial autophagy synergizing with SOR to achieve a dual ability to inhibit HCC activity (Tian et al., 2022). Metal-organic framework compounds MIL-101(Fe) NPs are capable of drug loading, controlled release, peroxidase activity, biocompatibility, and T2 magnetic resonance imaging. MIL-101(Fe)@sor NPs can increase lipid peroxidation and MDA levels, decrease GSH and GPX4, and inhibit tumor progression through ferroptosis (Liu et al., 2021). The NF-κB-specific promoter Cas13a or microRNA can selectively downregulate genes related to iron metabolism, fpn or lcn2. This in combination with iron nanoparticles can significantly induce ferroptosis (Sun et al., 2021).

It has also been shown that LDL nanoparticles and exosomes play a key role in the induction of ferroptosis to inhibit the growth of liver cancer cells. For example, LDL nanoparticles reconstituted from (LDL-DHA) natural omega-3 fatty acid, docosahexaenoic acid, significantly induce lipid peroxidation, GSH depletion, and inhibition of GPX4 activity. A study showed no association with apoptosis, necrosis or autophagy pathways from human HCC cell results. *In vivo*, elevated levels of lipid peroxidation and inhibition of GPX4 expression were found in liver tumor tissues, verifying the induction of ferroptosis by LDL-DHA (Ou et al., 2017). Exosomes are small membrane vesicles containing complex RNA and proteins. Small nanocapsules are novel communication and drug delivery mediators that can transport biologically active molecules between cells *via* a variety of biomolecules (e.g., proteins, nucleic acids) and regulate the cellular microenvironment and immune system (Chen R et al., 2019). Since exosomes are susceptible to phagocytosis by the monocyte phagocytic system (MPS), functionalization of CD47 on exosomes (ExosCD47) effectively avoids the phagocytic effect of MPS. This facilitates loading of Erastin and photosensitizer (Rose Bengal, RB) into exosomes to inhibit HCC viability by targeting induction of ferroptosis through chemophotodynamic therapy (Du et al., 2021). In summary, novel technologies such as nanotechnology and chemophotodynamic therapy have the potential to target and improve HCC treatment, enhance the ability of drugs to induce ferroptosis, and provide new possibilities for development of new drug treatment vectors.

### Others

In addition to the above related drug studies, there is clear evidence that Erastin, sulfasalazine (SAS) can also inhibit HCC activity by inducing ferroptosis. For example, microRNA-214-3p (miR-214) further increases MDA and ROS expression levels, upregulates Fe^2+^ concentration and decreases GSH levels when combined with Erastin. This is mainly due to Erastin mediated upregulation of activating transcription factor (ATF4). miR-214 ameliorates this upregulation, and the combination reduces the ability of ATF4 to inhibit ferroptosis, thus upregulating ferroptosis (Bai et al., 2020). Ketamine (a derivative of phencyclidine) inhibits GPX4 expression by reducing plasmacytoma variable translocation gene 1 (lncPVT1), and lncPVT1 promotes GPX4 expression by adsorbing miR-214-3p. The LncPVT1/miR-214-3p axis is one of the potential mechanisms by which ketamine regulates GPX4 expression to modulate iron sagging in HCC cells (Zhu et al., 2021). Haloperidol (Haloperidol), a typical butylphenyl antipsychotic, has been reported in recent years to have a high affinity for Sigma-1 (S1R) (Rousseaux and Greene, 2016). Downregulation of S1R can generate oxidative stress (Wang et al., 2015; Bai et al., 2019). Haloperidol treatment significantly downregulates GSH levels and upregulates lipid peroxidation levels in HCC cells (Bai et al., 2017). The nuclear protein 1 (NUPR1) inhibitor ZZW-115 is able to disrupt mitochondrial morphology and metabolic function by inhibiting the expression of mitochondrial transcription factor A (TFAM). This leads to accumulation of lipid peroxidation, thus inducing ferroptosis (Huang et al., 2021).

Additional partial studies that blocked ferroptosis in HCC enabled a better reverse understanding of the underlying mechanisms of escape resistance in cancer. For example, YAP/TAZ induces the expression of SLC7A11 in a TEAD-dependent manner. By maintaining the protein stability, nuclear localization and transcriptional activity of the transcriptional activator factor (ATF4), HCC cells inhibit SOR-induced ferroptosis (Gao et al., 2021). In addition, Necrostatin-1 inhibits System xc^−^ mediated ferroptosis in Huh7 and SK-HEP-1 cells probably by inducing xCT expression (Yuk et al., 2021).

In conclusion, the inhibition of ferroptosis in HCC has great therapeutic potential. Such inhibition involves direct inducers of ferroptosis such as SOR, Erastin, SAS, etc., which can act synergistically or antagonistically to ferroptosis inducers by inducing or inhibiting the activity of related targets. The above also describes the role of natural products in the induction of ferroptosis inhibitory activity in HCC in recent years, revealing the potential value of natural products in the inhibition of HCC. In addition, rapid development of new technologies such as nano- and exosomes have a high potential in facilitating inhibition of HCC. The review of modern drug-induced ferroptosis inhibition of HCC viability can be a guide for subsequent research and development targeting ferroptosis inhibition of HCC.

### Prognosis of ferroptosis in hepatocellular carcinoma

Prognostic analysis can be clinically important in predicting the progression of disease after onset. Prognostic analysis of HCC and ferroptosis will provide further insight into the induction of ferroptosis to inhibit HCC. Numerous studies have shown that ACSL4,SL7A11,SLC3A2, and G6PD are major regulators in ferroptosis. ACSL4 (Du and Zhang, 2020; Feng J et al., 2021), SL7A11 (Tang B et al., 2020; Zhang et al., 2021), SLC3A2, and G6PD (Dai et al., 2021) are genes associated with ferroptosis in HCC with clear prognostic significance. ABCB6 (Zhang et al., 2020), UBA1 (Shan et al., 2020) etc., have good predictive ability in HCC and ferroptosis by affecting HO-1, FTH1, FTL to regulate iron metabolism and induce ferroptosis. Some long-stranded non-coding RNAs (IncRNAs) also have good predictive ability in ferroptosis-induced HCC (Chen Z. A et al., 2021; Xu et al., 2021). In summary, modern prognostic analysis in ferroptosis of HCC using bioinformatics tools is important for finding key targets for treatment.

## Conclusion

Ferroptosis is an important form of regulatory cell necrosis. Proper induction or inhibition of cellular ferroptosis can help improve and treat a variety of diseases. Ferroptosis plays a very important role in HCC. Currently, the main drugs that induce ferroptosis in HCC are SOR, SAS, etc., Natural product extracts and monomers including Chinese herbs also provide new strategies for HCC treatment. To further improve drug resistance, nanotechnology, and drug combination therapy in the induction of HCC ferroptosis is a promising research hotspot. Ferroptosis-targeting drugs, drug combination applications, nanotechnology, etc., act to induce ferroptosis in HCC mainly through System xc^−^/GSH/GPX4, iron metabolism, p53, and lipid peroxidation pathways. These signaling pathways intersect with each other and exert combined effects. A large number of bioinformatics studies for prognostic analysis of induced HCC ferroptosis provide information not only for the study of new targets in HCC but also to support better clinical treatment.

Not surprisingly, the role of ferroptosis in HCC has attracted the interest of clinical researchers. Ferroptosis has a complex interdependent role in liver tumor prevention, diagnosis, prognosis, and treatment. These aspects will require extensive and ongoing research to better understand the regulatory mechanisms and signaling pathways of ferroptosis in HCC. In recent years, TCM has emerged to have a clear role in suppressing liver tumors. The TCM system is huge, but there are a few reports on induction of ferroptosis to inhibit HCC by TCM, which holds great promise in future HCC research. Solving the global problem of liver cancer patients through TCM alone or in combination with clinical western drugs to induce ferroptosis is a challenge. Therefore, we believe that induction of ferroptosis in HCC either by TCM alone or in combination with modern techniques will provide a better strategy to improve the treatment and prognosis of HCC.

## Abbreviations

HCC, HCC; GSH, Glutathione; ACD, Accidental cell death; RCD, Regulated cell death; RR, Ribonucleotide reductase; FUF, Ferroptosis upregulation factor; FDF, Ferroptosis downregulation factor; System xc^-^, Cystine/Glutamate transporter; x CT, SLC7A11; TRIB2, Tribbles homolog 2; CP, Copper cyanidin; PUFAs, Polyunsaturated fatty acids; G6PD, Glucose-6-phosphate dehydrogenase; SOR, Sorafenib; FTH1, Heavy chain ferritin; HMOX1, Heme oxygenase 1; ROS, Reactive oxygen species; MT1, Metallothionein-1; GSTZ1, Glutathione S-transferase zeta 1; Rb, Retinoblastoma; DHA, Dihydroartemisinin; FC, Formosanin C.
